# Type 2 diabetes mellitus’ impact on heart failure patients’ exercise tolerance: a focus on maximal fat oxidation during exercise

**DOI:** 10.3389/fcvm.2025.1485755

**Published:** 2025-02-10

**Authors:** Huiying Zhu, Jianchao Pan, Jianxuan Wen, Xiaojing Dang, Xiankun Chen, Yunxiang Fan, Weihui Lu, Wei Jiang

**Affiliations:** ^1^ The Second Affiliated Hospital of Guangzhou University of Chinese Medicine, Guangzhou, China; ^2^ Department of Cardiology, Guangdong Provincial Hospital of Chinese Medicine, Guangzhou, China; ^3^ The Second Clinical Medical College of Guangzhou University of Chinese Medicine, Guangzhou, China; ^4^ Department of Endocrinology, Guangdong Provincial Hospital of Chinese Medicine, Guangzhou, China; ^5^ Key Unit of Methodology in Clinical Research, Guangdong Provincial Hospital of Chinese Medicine, Guangzhou, China; ^6^ Heart Failure Center/Department of Cardiology, Guangdong Provincial Hospital of Chinese Medicine, Guangzhou, China

**Keywords:** heart failure, type 2 diabetes mellitus, exercise tolerance, maximal fat oxidation, fat oxidation

## Abstract

**Objective:**

To explore the impact of type 2 diabetes mellitus (T2DM) on exercise tolerance and fat oxidation capacity in patients with heart failure (HF).

**Methods:**

We retrospectively analyzed 108 Chinese patients with HF who were divided into a diabetic group (T2DM group, *n* = 47) and a non-diabetic group (non-T2DM group, *n* = 61). All subjects completed cardiopulmonary exercise testing (CPX). We determined their fat oxidation (FATox) by indirect calorimetry.

**Results:**

In the HF patients, the peak oxygen uptake (VO_2_) value was 14.76 ± 3.27 ml/kg/min in the T2DM group and 17.76 ± 4.64 ml/kg/min in the non-T2DM group. After adjusting for age, sex, body mass index (BMI), log N-terminal pro-B type natriuretic peptide (log NT-proBNP), left ventricular ejection fraction (LVEF), hemoglobin, renal function, coronary heart disease and hypertension, the peak VO_2_ was lower in the T2DM group compared to the non-T2DM group with a mean difference (MD) of −2.0 ml/kg/min [95% confidence interval (CI), −3.18 to −0.82, *P* < 0.01]. The VO_2_ at anaerobic threshold (AT VO_2_) was also lower in the T2DM group than in the non-T2DM group, with a MD of −1.11 ml/kg/min (95% CI −2.04 to −0.18, *P* < 0.05). Regarding the fat oxidation capacity during CPX, the T2DM group's maximal fat oxidation (MFO) was lower than that of the non-T2DM group (0.143 ± 0.055 vs. 0.169 ± 0.061 g/min, *P* < 0.05). In addition, the T2DM group had lower FATox at exercise intensity levels of 40% (*P* < 0.05) and 50% (*P* < 0.05) of peak VO_2_, compared to the non-T2DM group.

**Conclusions:**

T2DM is associated with a decrease in exercise tolerance and fat oxidation capacity in patients with heart failure. Thus, it could be useful to develop exercises of appropriate intensity to optimize physical and metabolic health.

## Introduction

At least 64 million people worldwide are affected by heart failure (HF), and its prevalence is increasing (1). Type 2 diabetes mellitus (T2DM) is common in HF patients, and increases both their mortality and morbidity (2). Patients with HF and T2DM exhibit specific metabolic, neurohormonal disorders and cardiac structural abnormality, which independently exacerbate adverse patient outcomes, quality of life, and costs of care. Therefore, much emphasis has been placed on the clinical evaluation and intervention of HF patients with T2DM.

Exercise intolerance is manifested either by decreased peak workload or peak oxygen uptake (VO_2_), which is a powerful marker of impaired health status. A low level of peak VO_2_ is known to be a strong predictor of all-cause mortality in HF patients, and it also indicate the disease severity and unfavorable prognosis of individuals with HF (3). The peak VO_2_ in HF patients is affected primarily by central factors such as cardiac function, pulmonary function, vascular function, and peripheral factors such as skeletal muscle function. A variety of skeletal muscle disorders are common in HF patients, and help explain exercise intolerance. A recent study has shown that reduced fat oxidation (FATox) was present in the skeletal muscle of HF patients, and was an independent factor for predicting reduced muscle endurance (4).

In terms of exercise tolerance in patients with T2DM, a reduction of approximately 20%–30% in peak VO_2_ can be observed even in the absence of overt cardiovascular or pulmonary disease (5). Furthermore, studies have investigated T2DM's detrimental impact on exercise capacity in HF patients (6). The mechanism may be related to abnormal energy metabolism.In heart failure, there is a shift in myocardial metabolism from glucose oxidation to fatty acid oxidation. This shift is often associated with a decrease in the efficiency of energy production, as fatty acid oxidation requires more oxygen to produce the same amount of ATP compared to glucose oxidation. This metabolic inefficiency can lead to reduced exercise tolerance in HF patients (7). Diabetes and insulin resistance can reduce the insulin-mediated low-oxygen glucose energy supply and increase the reliance of the myocardium and skeletal muscles on fatty acids, thereby exacerbating the decline in exercise tolerance in heart failure (8, 9). Interestingly, individuals with T2DM exhibit skeletal muscle abnormalities akin to those seen in HF patients, including impaired energy metabolism, alterations in muscle fiber types, and mitochondrial dysfunction (5). Consequently, T2DM may exacerbate exercise intolerance among HF patients by influencing skeletal muscle metabolic function.

Adipose tissue serves as a crucial energy reservoir, facilitating sustained and stable energy supply to the body through oxidative processes. FATox predominantly occurs within the mitochondria of skeletal muscle cells. Typically, low to moderate intensity aerobic exercise relies on FATox as a primary energy source, underscoring the indispensability of efficient fat oxidation capacity in facilitating daily activities. Moreover, FATox plays a pivotal role in regulating endocrine homeostasis, and is involved in antioxidant defense mechanisms and immune modulation processes.

Energy substrate utilization indicates that FATox gradually increases from low to moderate exercise intensity, peaking at a certain intensity and then declining. The highest FATox value during exercise is referred to as maximal fat oxidation (MFO). The exercise intensity eliciting MFO (Fat_max_) has been reported to be between 47% and 75% of VO_2max_ (10). Defective fatty acid oxidation, intramyocellular lipid accumulation and altered mitochondrial energetics are all associated with insulin resistance and T2DM (11). The decrease in metabolic substrate oxidation utilization during exercise will affect the production and conversion of energy, resulting in exercise intolerance or premature end of the exercise test. However, few studies have investigated patients with HF and T2DM from the perspective of exercise tolerance and FATox. The aim of this study is to investigate T2DM's effect on exercise tolerance and fat oxidation capacity in patients with HF.

## Methods

### Patients

**Figure 1:**
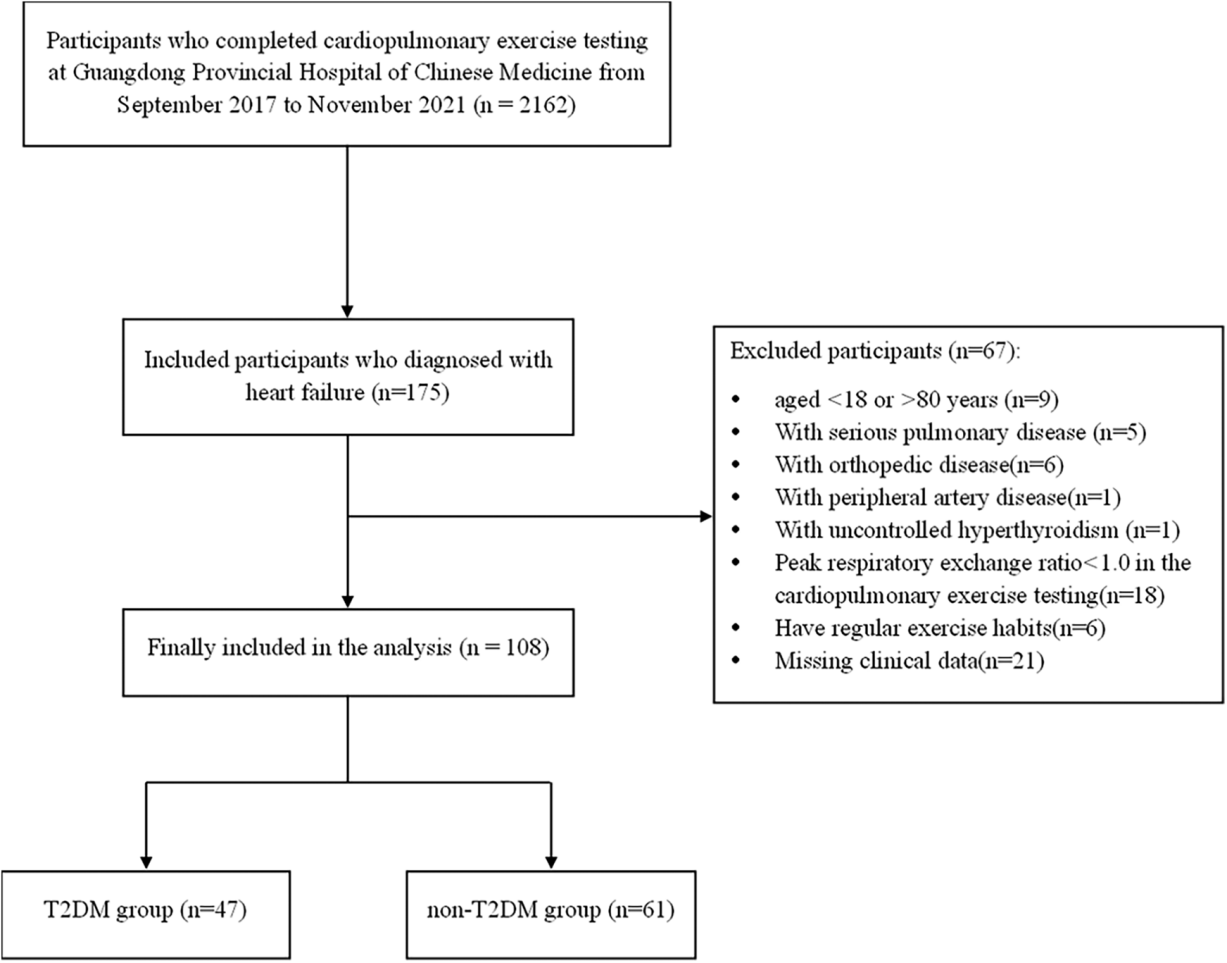
Flow chart for patient selection. T2DM: type 2 diabetes mellitus.

We retrospectively analyzed the cases of a total of 108 sedentary Chinese patients with HF who had undergone cardiopulmonary exercise testing (CPX) at Guangdong Provincial Hospital of Chinese Medicine, from September 2017 to November 2021. All of the patients had been diagnosed with HF based on the American College of Cardiology Foundation (ACCF)/American Heart Association (AHA) Task Force on Practice guidelines (12), and were from 18 to 80 years of age. We excluded any patients with a history of serious pulmonary disease, peripheral artery disease, orthopedic disease or endocrine disorder other than T2DM. We also excluded any patients whose peak respiratory exchange ratio (RER) did not reach 1.0 in the CPX, as they might not be able to achieve MFO. The patients were not treated with any hormonal substitutions. The subjects were categorized into the following two groups: patients with HF and T2DM (the T2DM group; *n* = 47) and patients with HF without T2DM (the non-T2DM group; *n* = 61). Patients in the T2DM group were diagnosed with type 2 diabetes mellitus by a diabetologist before the CPX. The flow chart of patient selection is shown in Figure 1.

This study was conducted in accordance with the Declaration of Helsinki, and has been approved by the ethics committee at the Guangdong Provincial Hospital of Chinese Medicine (number: ZE2019-217-01). Prior to the CPX, all subjects were informed about the study procedures, the exercise protocol and possible risks and benefits, and voluntarily signed the written informed consent form.

### Cardiopulmonary exercise testing

Before each test, the ambient reference was checked, and volume and gas concentration were calibrated. All participants were instructed to refrain from eating 2 h before the test, and to abstain from caffeine and alcohol, but water was allowed. Subjects were also told to avoid any physical activity on the day of the test. They were instructed to sit and rest for at least 30 min before the exercise test, and then the resting heart rate and blood pressure were measured. Meanwhile, the standard exercise testing procedures were explained to the subjects.

All subjects performed the CPX on an upright cycle ergometer (CS-200 Ergo-Spiro, SCHILLER, Baar, Switzerland) using a ramp protocol (7–16 watts/min). They were told to warm up with 0 watts for 3 min. After the warm-up period, subjects were verbally encouraged to maintain the desired pedal rate of 60 rpm and to exercise until volitional exhaustion or until they presented the termination criteria. The exercise tests were finished within 6–12 min. Heart rate (HR), arrhythmia and ST-T wave changes were monitored continuously, and blood pressure was measured every minute during exercise. Respiratory gas analysis was performed with a breath-by-breath apparatus (Power Cube; Ganshorn, Niederlauer, Germany) and LF8 software (CARDIOVIT, CS-200 ergospirometry; SCHILLER). The following data were collected automatically from the system: HR (beat/min), oxygen consumption (VO_2_, ml/kg/min and ml/min), carbon dioxide production (VCO_2_, L/min), respiratory exchange ratio (RER = VCO_2_/VO_2_), metabolic equivalents (METS), ventilatory equivalents for carbon dioxide (VE/VCO_2_ slope) and oxygen pulse (O_2_ pulse, ml/beat). The anaerobic metabolic threshold (AT) was determined according to the V-slope method (13). Peak VO_2_ was calculated as the average of the values obtained during the last 30 s of the exercise. O_2_ pulse was calculated as VO_2_/HR.

### Fat oxidation rate measurements

We used indirect calorimetry to quantify the fat oxidation rate. VO_2_ and VCO_2_ were continuously collected and averaged over 10 s throughout the entire test. The dates of VO_2_ and VCO_2_ were used to calculate FATox over the exercise intensity range for each subject by using the Péronnet and Massicotte's equation (14): FATox (mg·min^−1^) = 1.695·VO_2_ (L·min^−1^)−1.701·VCO_2_ (L·min^−1^). The MFO and the FATox at 20%–60% VO_2max_ were calculated and recorded.

### Other clinical variables and outcomes

Patients’ demographic and clinical data were obtained through their medical records, including age, sex, body mass index (BMI), New York Heart Association (NYHA) functional classification, primary disease (coronary heart disease, myocardial infarction, cardiomyopathy, valvular disease, atrial fibrillation, atrial flutter), diabetes duration, and the presence of any comorbidity (hypertension, dyslipidemia, stroke, chronic kidney disease, chronic obstructive pulmonary disease). Items to be researched by biochemical blood examination included HbA1c, fasting plasma glucose, N-terminal pro brain natriuretic peptide (NT-proBNP), high-sensitivity C-reactive protein (hsCRP), hemoglobin, serum creatinine and estimated glomerular filtration rate (eGFR). Echocardiographic indexes included left ventricular ejection fraction (LVEF), left ventricular end-diastolic dimension (LVEDD), pulmonary arterial systolic pressure (PASP) and stroke volume (SV). Medication history to be researched included oral drugs used (angiotensin converting enzyme inhibitor, angiotensin receptor blockers, angiotensin receptor enkephalinase inhibitor, β blocker, mineralocorticoid receptor antagonists, statins, diuretics, calcium antagonists, antiplatelet agents, anticoagulant and oral hypoglycemic agent) and the use of insulin. The above datapoints, such as the echocardiographic parameters, laboratory data, and medication were acquired within the 2 weeks before or after CPX examination.

### Statistical analyses

Continuous variables were presented as means with standard deviation (SD). Categorical variables were reported as counts (percentages). Baseline demographic and clinical characteristics of the two groups were compared using either a Student's *t*-test or a Mann–Whitney *U* test, as appropriate. Count data and grade data were analyzed using a chi-square test and Fisher's exact test. Multivariate analysis was conducted using multiple linear regression.

Based on literature reports, age, sex, BMI, log NT-proBNP, LVEF, hemoglobin, eGFR, history of coronary heart disease, hypertension, hyperlipidemia, and statin use were identified as confounding variables. The CPX parameters were compared between the two groups after adjustment for age, sex, BMI, log NT-proBNP, LVEF, hemoglobin, eGFR, history of coronary heart disease and hypertension, all of which are considered confounders. The maximal fat oxidation parameters were compared between the two groups after adjusting for age, sex and BMI. FATox between the two groups during exercise was compared using a Student's *t*-test. A confidence interval was set at 95%. *p* < 0.05 was considered statistically significant. All statistical procedures were performed in SPSS (version 19.0, Chicago, IL, USA).

## Results

### HF patients’ baseline characteristics

The baseline clinical characteristics of the T2DM group (*n* = 47) and the non-T2DM group (*n* = 61) are summarized in Table 1. The mean age of the total HF population was 60.9 ± 11.4 years, and 82% were men. The T2DM duration was 6.7 ± 6.8 years. A significant difference was observed between the two groups in terms of HbA1c, fasting plasma glucose, the prevalence of coronary heart disease and hypertension, statin and antiplatelet agent drug usage (*P* < 0.05). There was no significant difference in any other basic attributes, primary disease status, comorbidity, or medication history, between the two groups.

**Table 1:** HF patients’ baseline characteristics.

	All (*n* = 108)	Diabetic (*n* = 47)	Non-diabetic (*n* = 61)	*P*-value
Demographics				
Age, years	60.9 ± 11.4	62.6 ± 9.1	59.5 ± 12.8	0.239
Male, *n*	89 (82)	40 (85)	49 (80)	0.518
BMI, kg/m^2^	24.7 ± 3.6	24.8 ± 3.5	24.7 ± 3.7	0.795
T2DM duration, years	–	6.7 ± 6.8	–	NA
NYHA class				
NYHA Ⅰ, *n*	10 (9)	3 (6)	7 (12)	0.501
NYHA Ⅱ, *n*	60 (56)	25 (53)	35 (57)	
NYHA Ⅲ, *n*	38 (35)	19 (41)	19 (31)	
Comorbidity				
Coronary heart disease, *n*	73 (68)	38 (81)	35 (57)	0.010*
Myocardial infarction, *n*	33 (31)	17 (36)	16 (26)	0.266
Cardiomyopathy, *n*	30 (28)	11 (23)	19 (31)	0.373
Valvular disease, *n*	46 (43)	18 (38)	28 (46)	0.428
Atrial fibrillation/flutter, *n*	26 (24)	10 (21)	16 (26)	0.551
Hypertension, *n*	62 (57)	34 (72)	28 (46)	0.006*
Hyperlipoidemia, *n*	42 (39)	20 (43)	22 (36)	0.493
Stroke (ischemic), *n*	7 (7)	4 (9)	3 (5)	0.466
Chronic kidney disease, *n*	12 (11)	8 (17)	4 (7)	0.086
Chronic obstructive pulmonary disease, *n*	5 (5)	3 (6)	2 (3)	0.651
Echocardiographic findings				
LVEF, %	49.2 ± 11.5	47.6 ± 11.0	50.4 ± 11.9	0.157
LVEDD, mm	56.0 ± 8.4	56.1 ± 7.4	55.9 ± 9.2	0.515
PASP, mmHg	30.2 ± 9.4	30.0 ± 11.5	30.4 ± 7.7	0.311
SV, ml/bit	73.7 ± 24.2	75.7 ± 23.0	72.4 ± 25.0	0.542
Laboratory measurements				
NT-proBNP, pg/L	977.8 ± 1,326.9	1,198.3 ± 1,779.2	806.6 ± 799.3	0.151
hsCRP, mg/L	4.7 ± 6.1	5.3 ± 6.0	4.2 ± 6.3	0.104
Hemoglobin, g/L	141.2 ± 17.4	138.8 ± 17.8	143.0 ± 17.1	0.221
Serum creatinine, mg/dl	104.3 ± 44.4	114.1 ± 62.6	96.7 ± 18.9	0.306
eGFR, ml/min/1.73 m^2^	70.2 ± 20.6	67.1 ± 24.3	72.7 ± 16.9	0.212
Fasting plasma glucose	7.0 ± 2.2	8.2 ± 2.4	6.0 ± 1.6	0.000*
HbA1c, %	6.7 ± 1.3	7.3 ± 1.4	5.8 ± 0.5	0.000*
Current medications				
ACEI/ARB, n	54 (50)	23 (49)	31 (51)	0.846
ARNI, *n*	32 (30)	13 (28)	19 (31)	0.694
Beta-blockers, *n*	98 (91)	44 (94)	54 (89)	0.568
MRAs, *n*	58 (54)	22 (47)	36 (59)	0.207
Statins, *n*	85 (79)	44 (94)	41 (67)	0.001*
Diuretics, *n*	37 (34)	16 (34)	21 (34)	0.967
Calcium antagonists, *n*	15 (14)	9 (20)	6 (10)	0.165
Antiplatelet agents, *n*	53 (49)	31 (66)	22 (36)	0.002*
Anticoagulant, *n*	35 (32)	12 (26)	23 (38)	0.180
Insulin, *n*	–	11 (23)	–	NA
SGLT-2, *n*	–	9 (19)	–	NA
Metformin, *n*	–	17 (36)	–	NA
DPP4 inhibitors, *n*	–	10 (21)	–	NA
Sulfonylureas, *n*	–	3 (6)	–	NA
Acarbose, *n*	–	14 (30)	–	NA
Glinides, *n*	–	5 (11)	–	NA

Data are presented as mean ± SD or *n* (%). BMI, body mass index; NYHA, New York Heart Association; LVEF, left ventricular ejection fraction; LVEDD, left ventricular end-diastolic diameter; PASP, pulmonary artery systolic pressure; SV, stroke volume; NT-proBNP, N-terminal pro-B type natriuretic peptide; hsCRP, high-sensitivity C-reactive protein; eGFR, estimated glomerular filtration rate; HbA1c, glycohemoglobin A1c; ACEI/ARB, angiotensin-converting enzyme inhibitors/angiotensin receptor blocker; MRAs, mineralocorticoid receptor antagonists; SGLT-2, sodium-dependent glucose transporters 2, DPP4, dipeptidyl peptidase-4; NA, not applicable; *P < 0.01 vs. non-diabetic.

### HF patients’ exercise capacity

The CPX data for the HF patients are summarized in Table 2. Peak VO_2_ was a major indicator reflecting maximum exercise capacity. The mean peak VO_2_ value was 14.76 ± 3.27 ml/kg/min in the T2DM group and 17.76 ± 4.64 ml/kg/min in the non-T2DM group. The results of the multivariate analysis revealed that after adjusting for age, sex, BMI, log NT-proBNP, LVEF, hemoglobin, renal function, coronary heart disease and hypertension, the peak VO_2_ was lower in the T2DM group compared to the non-T2DM group, with a mean difference (95% CI) of −2.0 (−3.18 to −0.82) ml/kg/min (*P* < 0.01). AT VO_2_ was a sign of the body's submaximal aerobic capacity, which is not affected by patients’ subjective effort. After adjusting for age, sex, BMI, log NT-proBNP, LVEF, hemoglobin, renal function, coronary heart disease and hypertension, the AT VO_2_ was lower in T2DM group than in the non-T2DM group, with a mean difference (95% CI) of −1.11 (−2.04 to −0.18) ml/kg/min (*P* < 0.05).

**Table 2:** HF patients’ cardiopulmonary exercise testing parameters.

	All (*n* = 108)	Diabetic (*n* = 47)	Non-diabetic (*n* = 61)	Unadjusted mean difference (95% CI)	Adjusted mean difference (95% CI)
Peak VO_2_, ml/kg/min	16.45 ± 4.35	14.76 ± 3.27	17.76 ± 4.64	−3.00** (−4.57 to −1.42)	−2.00** (−3.18 to −0.82)
Peak VO_2_, L/min	1.12 ± 0.35	1.02 ± 0.28	1.20 ± 0.38	−0.18** (−0.31 to −0.05)	−0.11* (−0.20 to −0.02)
AT VO_2_, ml/kg/min	11.28 ± 2.64	10.41 ± 2.13	11.96 ± 2.80	−1.55** (−2.52 to −0.57)	−1.11* (−2.04 to −0.18)
Rest VO_2_, ml/kg/min	3.72 ± 0.63	3.64 ± 0.58	3.79 ± 0.66	−0.14 (−0.38 to 0.10)	−0.08 (−0.33 to 0.17)
Peak METS	5.02 ± 3.53	4.22 ± 0.94	5.64 ± 4.54	−1.43* (−2.76 to −0.09)	−1.74* (−3.22 to −0.26)
Peak workload, watts	88.78 ± 28.63	84.21 ± 24.6	92.3 ± 31.12	−8.08 (−19.04 to 2.88)	−4.09 (−11.04 to 2.85)
Peak RER	1.13 ± 0.06	1.14 ± 0.06	1.13 ± 0.07	0.01 (−0.01 to 0.04)	0.00 (−0.03 to 0.02)
Peak HR, beats min	123.12 ± 22.63	118.30 ± 17.97	126.84 ± 25.16	−8.54 (−17.13 to 0.05)	−5.55 (−14.59 to 3.5)
Peak SBP, mmHg	168.74 ± 34.09	162.77 ± 38.52	173.34 ± 29.75	−10.58 (−23.60 to 2.44)	−11.79 (−26.44 to 2.86)
Peak DBP, mmHg	89.34 ± 19.85	87.23 ± 19.53	90.97 ± 20.10	−3.73 (−11.37 to 3.91)	−3.83 (−11.79 to 4.14)
Peak O_2_ pulse, ml/beats	9.18 ± 2.61	8.66 ± 2.42	9.58 ± 2.70	−0.92 (−1.91 to 0.08)	−0.69 (−1.49 to 0.11)
VE/VCO_2_ slope	32.95 ± 6.77	34.87 ± 6.83	31.48 ± 6.40	3.40** (0.86 to 5.93)	2.35 (−0.23 to 4.92)

Data are presented as mean ± SD. Mean difference between the diabetic and the non-diabetic is adjusted by age, sex, BMI, LVEF, log NT-proBNP, hemoglobin, eGFR, coronary heart diseases and hypertension. AT, anaerobic threshold; RER: respiratory exchange ratio; HR, heart rate; SBP, systolic blood pressure; DBP, diastolic blood pressure; VO_2_, oxygen uptake; VCO_2_, carbon dioxide production; VE, minute ventilation; 95% CI, 95% confidence interval; *P < 0.05 vs. non-diabetics; **P < 0.01 vs. non-diabetics.

### Fat oxidation during HF patients’ exercise

**Figure 2:**
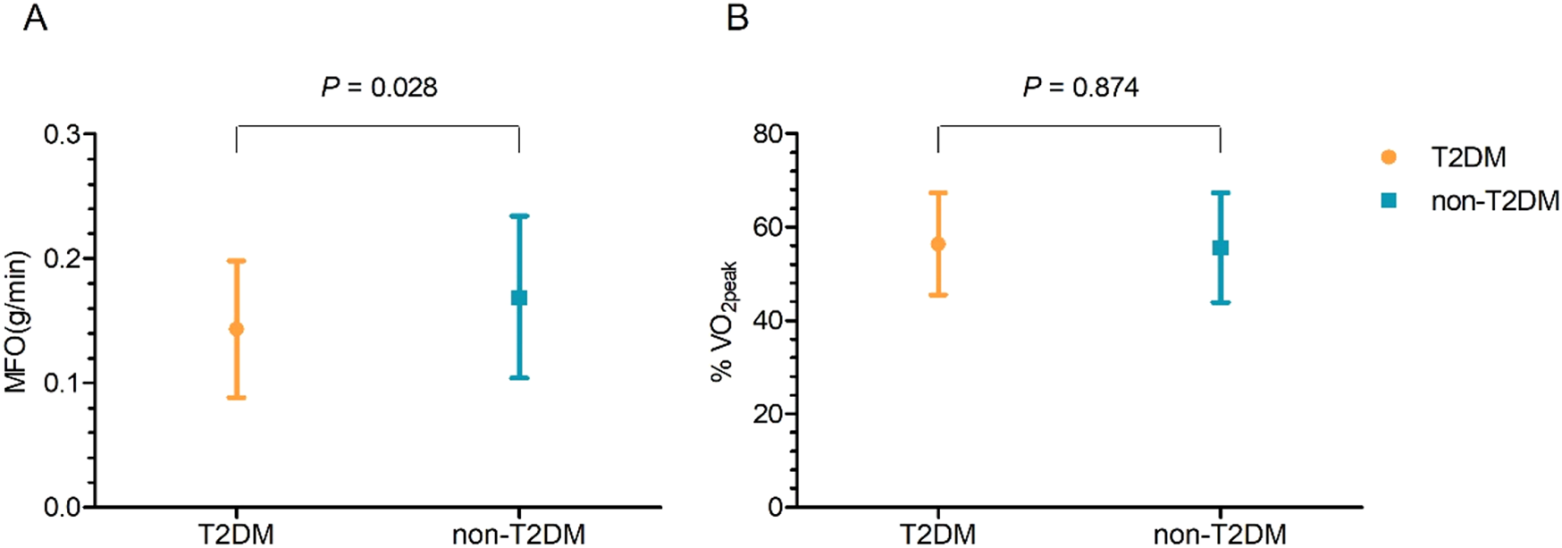
Maximal fat oxidation (MFO) comparison **(A)** and peak VO_2_ (%VO_2peak_) percentage at the MFO **(B)** date are means ± SD.

**Figure 3:**
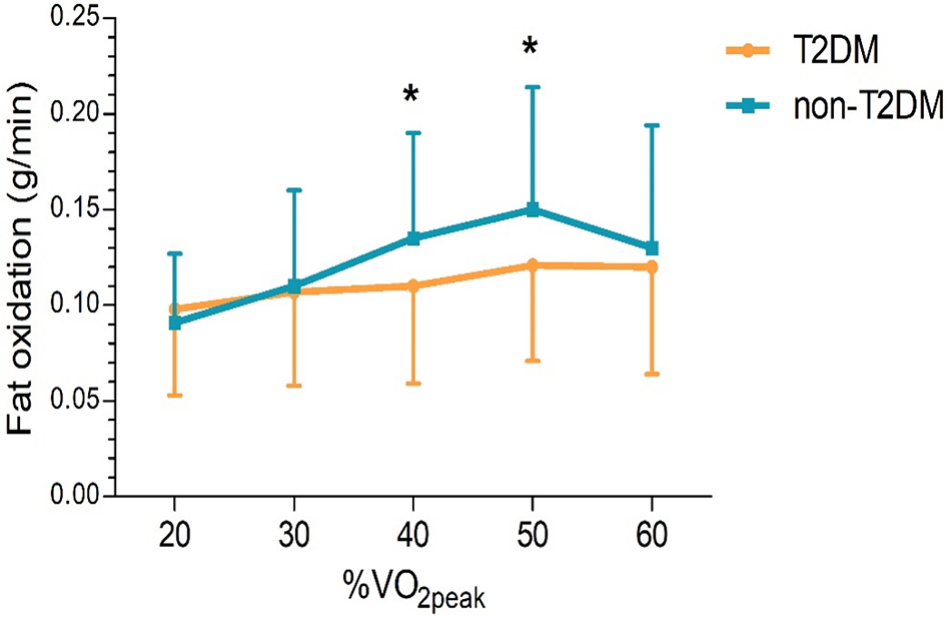
Fat oxidation rates, according to peak VO_2_ percentage (%VO_2peak_). The dates are means ± SD. *P < 0.05 vs. non-diabetics.

The two groups’ fat oxidation parameters are shown in Figure 2; Table 3. During the exercise test, the T2DM group's MFO was significantly lower than that of the non-T2DM group (0.143 ± 0.055 vs. 0.169 ± 0.061 g/min, *P* < 0.05; Figure 2A). The results of the multivariate analysis revealed that after adjusting for age, sex, BMI, LVEF, coronary heart diseases, hyperlipoidemia and statins, the MFO was lower in the T2DM group compared to the non-T2DM group, with a mean difference (95% CI) of −0.027 (−0.051 to −0.003) g/min (*P* < 0.05). Between the two groups, there was no difference in percentage of peak VO_2_ at the point of MFO (56.44 ± 10.93% vs. 55.63 ± 11.74%, *P* > 0.05; Figure 2B). During the exercise, the FATox as a function of the peak VO_2_ percentage are presented in Figure 3, Table 4. When looking at each intensity point, the T2DM group's FATox was significantly lower than that of the non-T2DM group when the intensity level was at 40% (*P* < 0.05) and 50% (*P* < 0.05) of peak VO_2_.

**Table 3:** HF patients’ maximal fat oxidation parameters.

	All (*n* = 108)	Diabetic (*n* = 47)	Non-diabetic (*n* = 61)	Unadjusted mean difference (95% CI)	Adjusted mean difference (95% CI)
MFO, g/min	0.157 ± 0.062	0.143 ± 0.055	0.169 ± 0.065	−0.026* (−0.049 to −0.003)	−0.027* (−0.051 to −0.003)
%VO_2peak_ at MFO, %	55.98 ± 11.35	56.44 ± 10.93	55.63 ± 11.74	−0.813 (−5.198 to 3.572)	−0.335(−4.501 to 3.831)

Data are presented as mean ± SD. Mean difference between the diabetic and the non-diabetic is adjusted by age, sex, BMI, LVEF, coronary heart diseases, hyperlipoidemia and statins. %VO_2peak_, percentage of peak VO_2_; 95% CI, 95% confidence interval; *P < 0.05 vs. non-diabetics.

**Table 4:** Fat oxidation (g/min) during intradermal exercise.

Intensity	Diabetic (n = 47)	Nondiabetic (*n* = 61)	Unadjusted mean difference (95% CI)	*P*-value
20%VO_2peak_	0.098 ± 0.045	0.091 ± 0.036	0.007 (−0.008 to 0.023)	0.353
30%VO_2peak_	0.107 ± 0.049	0.110 ± 0.050	−0.002 (−0.021to 0.017)	0.815
40%VO_2peak_	0.110 ± 0.051	0.135 ± 0.055	−0.025 (−0.046 to −0.005)	0.016*
50%VO_2peak_	0.121 ± 0.050	0.150 ± 0.064	−0.029 (−0.052 to −0.006)	0.012*
60%VO_2peak_	0.120 ± 0.056	0.130 ± 0.064	−0.009 (−0.034 to 0.016)	0.471

Data are presented as mean ± SD. %VO_2peak_, percentage of peak VO_2_; 95% CI, 95% confidence interval; *P < 0.05 vs. non-diabetics.

## Discussion

The present study's first finding was that both peak VO_2_ and AT VO_2_ in HF patients with T2DM were lower than those in patients without T2DM after adjusting for age, sex, BMI, log NT-proBNP, LVEF, hemoglobin, renal function, coronary heart disease and hypertension. Secondly, the FATox in the T2DM group was lower than that in the non-T2DM group during exercise. These results suggest that T2DM complications cause exercise intolerance and the dysregulation of fat metabolism during exercise in HF patients.

Exercise intolerance is widely recognized as HF's main clinical symptom. All patients with HF in this study completed exercise tests until they reached a peak VO_2_ without adverse effects (e.g., cardiovascular events, dyspnea, dizziness, or general malaise), or any exercise-induced hypoglycemia in the T2DM group. This study found that T2DM harms exercise tolerance in HF patients. Similar findings have been reported in previous studies, some of which have attempted to explore its mechanisms. T2DM detracts from exercise endurance in HF patients by affecting cardiovascular, pulmonary, neuroendocrine, metabolism, and skeletal muscle function, such as cardiac output, pulmonary volume, muscle blood distribution and diffusion, skeletal muscle aerobic capacity, and fatigue perception. A recent study has shown that T2DM is associated with lowered peak VO_2_ in HF patients, and the extent of the effect depends partly on left ventricular systolic function, as well as other peripheral non-cardiac factors (15). In addition, it has been reported that patients with HF and T2DM have a low heart rate response and abnormal sympathetic and parasympathetic nerve activities during CPX. This suggests that heart rate response attenuation due to DM-induced decreases in autonomic activity may be one mechanism of reduced exercise tolerance (16). Left ventricular insufficiency can lead to changes in pulmonary volumes and gas diffusion, and these pulmonary mechanisms contribute to exercise intolerance in HF patients. In HF patients with T2DM, abnormalities in the ventilatory pattern during exercise and greater impedance to alveolar-capillary gas transfer have been observed, aggravating pulmonary dysfunction and exercise intolerance (17). Meanwhile, DM increases the prevalence of peripheral vascular disease, and impairs endothelial function from oxidative stress, vasoconstriction, and inflammation, which reduces the difference in arterial-venous oxygen content, and affects exercise endurance (18). It should be noted that metabolic disorders and skeletal muscle dysfunction are the causes of exercise intolerance in T2DM patients. However, whether there is abnormal metabolic substrate oxidation in HF patients with T2DM has yet to be reported. Therefore, the present study investigated the effects of diabetes comorbidities on exercise endurance in patients with HF from the perspective of abnormal fat oxidation (FATox) during exercise.

Lipids are important energy supply substrates for muscle contraction, originating from subcutaneous adipose tissue, intramuscular triacylglycerides, cholesterol and dietary fat (19). When metabolic demand increases, free fatty acids (FFA) are rapidly released from adipose tissue, and then taken up and oxidized in tissues such as liver, kidney, skeletal and myocardial muscle. Under normal conditions, fatty acids are the main substrate for ATP production in the heart with 50%–70% of ATP being derived from fatty acid oxidation (20). The energy metabolic changes occurring in heart failure are generally accepted to include reductions in mitochondrial fatty acid oxidation (21). However, in diabetes, there is a notable increase in myocardial fatty acid uptake. This not only leads to enhanced fatty acid oxidation but also results in a significant accumulation of lipids within the myocardium (22, 23). The intramyocardial accumulation of lipid metabolites include long chain acyl CoAs, diacylglycerols (DAG), triacylglycerols(TAG), and/or ceramide, which are thought to account for cardiac myocyte apoptosis, myocardial fibrosis, impaired mitochondrial function, and ultimately to cardiac dysfunction, known as lipotoxicity (22, 24). Furthermore, during exercise, the energy metabolism of the myocardium experiences distinct alterations. At higher exercise intensities, carbohydrate oxidation, particularly from muscle glycogen, takes precedence. In contrast, at lower intensities, fat oxidation plays a more crucial role (25). In fact, as exercise intensity increases, the energy contribution of FATox decreases, and the body subsequently shifts to carbohydrate oxidation (CHOox) for maintenance. However, the increase in FATox during exercise is accompanied by a decrease in CHOox due to muscle glycogen conservation (26). Previous studies have explored the associations between exercise capacity and FATox. In healthy men, VO_2max_ is positively correlated with resting FATox, MFO and 24 h FATox (27, 28).

The fat oxidation capacity during exercise and the exercise intensity that causes MFO reflect the level of metabolic health. After controlling for age, sex, weight and training status, T2DM patients present an impaired FATox at any exercise intensity, and also a lower Fat_max_ (29). Moreover, the FATox during exercise is positively correlated with peak VO_2_ in T2DM patients (30). Several mechanisms may account for diabetes’ negative effects on FATox. First, the diminished availability of FFA may contribute to the diminished FATox. Due to increased fatty acid re-esterification in adipose tissue, plasma FFA circulating concentrations decrease in T2DM patients at baseline conditions, as well as during physical activity, resulting in reduced FATox (31). Second, fat metabolism disorder is due in part to reduced FFA transport and uptake capacity in tissues. Due to poor peripheral circulation in T2DM patients, a portion of the FFA generated by exercise-induced lipolysis cannot be released into circulation (32). Meanwhile, in patients with T2DM during the postabsorptive state and β-adrenergic stimulation, increased skeletal muscle lipolysis floods the muscles with FFA, thereby reducing the plasma-muscle concentration gradient (33). Third, the endocrine system is primarily responsible for regulating FATox. The hormonal mechanisms that promote lipid metabolism are mainly based on catecholamines, cortisol, and growth hormone; in contrast, insulin is inhibitory (10). Insulin resistance and hyperglycemia have been proposed as important factors that may inhibit muscle FFA utilization in T2DM (34).

Although fat oxidation disorder in metabolic disease has been extensively studied, it has rarely been reported in HF patients. Our data showed that the mean MFO of all the HF subjects was lower than that reported in previous studies (10). This demonstrates that subjects with HF exhibit fat metabolic abnormality, which has been clinically associated with exercise intolerance and skeletal muscle dysfunction. Interestingly, we also found the fat oxidation capacity declined in the T2DM group compared to the control group. The above findings suggest that in patients with HF, there may be fat metabolism disorders within skeletal muscle associated with T2DM comorbidities, as reflected by reduced exercise capacity.

Many factors play a significant role in the exercise tolerance and fat metabolism of heart failure patients. Research indicates that dietary and exercise habits, as well as diabetes medications, can affect the exercise tolerance and lipid metabolism changes in heart failure patients. Dietary patterns are associated with improved exercise tolerance. Studies have shown that the Mediterranean diet can increase the peak oxygen uptake (VO₂peak) by 10% in patients with type 2 diabetes mellitus (T2DM), likely through enhanced mitochondrial function and reduced systemic inflammation (35). Alternatively, It has beenrecently shown that high fat diets promote FAox and haveperformance enhancement capabilities (36). However, definitive conclusions regarding pre-exercise macronutrient dominant diets and exercise performance improvements are contingent on specific exercise applications (37) that are directed by exercise duration and intensity (38). Diabetes medications, including metformin, SGLT2 inhibitors, and glucagon-like peptide-1 (GLP-1) receptor agonists, each have unique mechanisms and impacts on exercise tolerance and fat oxidation. Metformin can improve exercise tolerance and the rate of fat oxidation in patients with impaired glucose tolerance (39). GLP-1 binds to the GLP-1 receptors on the surface of cardiomyocytes, upregulating glucose transporter 4 in cardiomyocytes, thereby enhancing glucose uptake by cardiomyocytes, improving glucose metabolism in ischemic myocardium, and ultimately improving cardiac function (40). This study is retrospective and cannot trace and determine the dietary and exercise habits of the included patients. Additionally, due to the insufficient sample size, a more detailed analysis of diabetes medications was not conducted. This may have some impact on the study results, and necessitating further prospective studies or larger sample observational studies.

Exercise training has been used as a specific therapy for patients with HF as well as T2DM. Individualized exercise intensity is necessary to achieve various goals, such as optimizing glycemic control, improving cardiorespiratory fitness, metabolic function and body composition. While continuous moderate intensity training and high intensity interval training have been wildly recommended for cardiovascular and T2DM patients, studies have indicated that Fat_max_ training is also helpful for patients with metabolic disease and sub-optimal physical fitness (41). According to recent studies, Fat_max_ training in older people with T2DM improves body composition, blood glucose, lipid profile and insulin resistance, decreases BMI and abdominal obesity, and increases VO_2max_, and physical mobility (42, 43). Although there is no evidence of health benefits from Fat_max_ training in HF patients, metabolic training has been shown to significantly improve metabolic health, which may have important longitudinal influence for HF prognosis. As a result, optimizing whole-body fat oxidation capacity may be a potential therapy for skeletal muscle dysfunction in patients with HF, especially in those with diabetes mellitus. This study found that MD has a negative impact on VO₂ and MFO in HF patients. In clinical practice, for patients with HF, there is often a greater focus on training aimed at improving cardiopulmonary endurance, while the importance of metabolic training is overlooked. For patients with comorbidities such as obesity and diabetes, who have metabolic disorders, metabolic training is also a good option. However, the optimal exercise intensity, training duration, and other aspects of the training program require further investigation through randomized controlled trials.

## Limitations of this study

This study is retrospective and may have multiple confounding factors that could affect the results. Although statistical methods were used to control for confounding factors and multivariate analysis was conducted, these limitations cannot be completely eliminated. For example, factors such as patients’ exercise and dietary habits and the use of diabetes medications were not fully accounted for. Therefore, further prospective studies or larger sample observational studies are needed to explore the impact of diabetes on lipid metabolism and exercise tolerance in heart failure, as well as the underlying mechanisms.

## Conclusions

In this study, we found that type 2 diabetes mellitus (T2DM) is associated with a decrease in exercise tolerance and fat oxidation capacity in patients with heart failure. in HF patients. Regular training with individualized exercise intensity can improve exercise tolerance and fat oxidation capacity. Therefore, we suggest that patients with HF and T2DM can optimize physical and metabolic health through appropriate exercise training.

## Data Availability

The original contributions presented in the study are included in the article/Supplementary Material, further inquiries can be directed to the corresponding author/s.
